# Medical History from the Earliest Times

**Published:** 1893-05-27

**Authors:** E. T. Withington


### Medical History from the Earliest Times.

By E. T. Withington, M.A., M.B. Oxon.

### LVI.—THE IATROMECHANICAL SCHOOL.

While the doctrine of the Iatrochemists, starting from its centre in the Netherlands, blended in the schools of France and Germany with the remains of Galenic and Paracelsic theories, there arose in Italy and England a rival medical system, based upon the more advanced and exact sciences of mechanics and mathematics. This school, variously called the iatromathematical, iatromechanical, iatrophysical, or physiatric, was a direct product of the new scientic spirit, and its most important precursor was Santoro Sanctorius, professor at Padua, and the friend and colleague of Galileo. Sanctorius passed much of his life seated in a chair-like weighing machine of his own invention, by means of which he discovered the so-called "insensible perspiration," and found to his amazement that this imperceptible loss exceeds in amount all the other excretions combined. He published the results of countless experiments on himself in his "Medicina Statica" (1614), a work which, though it naturally attributes an excessive value to the new discovery, was of great use in pointing out the hygienic importance of the skin, in encouraging the introduction of diaphoretic medicines, and, above all, in showing that the rules of exact science might be applied to some departments of physiology. He expresses a wish that every one, or at least every physician, could have a similar machine, for not only are changes in weight among the earliest signs of approaching illness, but by taking his meals in such a chair a person would be guarded against irregularity in the quantity of his food, which is one of the most fertile causes of disease.

Sanctorius further invented many ingenious instruments, in devising some of which he was doubtless assisted by his colleague, the famous professor of mathematics. Thus his thermometers, which he declares will be invaluable in cases of fever, are imitations of the air and water instrument invented by Galileo (1597), slightly altered in shape to adapt them for being held in the mouth or applied to the skin. So, too, his "pulsilogium" is an application of Galileo's principle of the pendulum to measure the rate and regularity of the pulse. But we also find a trocar and cannula, "by which an opening may be rapidly made in the windpipe when suffocation is imminent, especially in infants," and which may also be used for tapping the abdomen in dropsy; a vessel resembling a bronchitis kettle, for distributing steam, narcotic vapours, and cooling odours in the sick room; and, finally, cupping glasses, fitted with exhausting syringes, described and figured at least twenty years before Guericke is supposed to have invented the air pump. Sanctorius, however, had no idea of establishing a new system of medicine, but rather intended to give more exactness and stability to the old Galenic theories. He belonged, in short, to the pre-Harveian age, and he concealed his most ingenious suggestions in a work where few were likely to look for them, a commentary on the "Canon" of Avicenna. But his other book, the "De Medicinâ Staticâ," had a great effect, and when the discovery of the circulation showed that the most typical of vital processes might be compared to a hydraulic system, even sober-minded physicians began to fancy that the Cartesian doctrine that the body is a machine, and physiology a depart ment of physics, might be not unjustified.

The first attempt to solve the problems of life and disease on these principles was made by Borelli (1608-79), in his famous work, "On the Motion of Animals" and, as far as the title is concerned, it was a brilliant success. He showed that walking, swimming, flying, &c., are mechanical processes in which muscles and bones play the part of strings and levers, and that the forces used can be estimated mathematically. But when he proceeds to more abstruse physiological and medical questions, he is less fortunate, and, like Sylvius, bases his whole system upon an error. Borelli declares that muscles, when in action, are increased in bulk, and that their activity is due to a rapid fermentation or explosion caused by the mixture of a drop of nerve fluid with the blood in the muscle. "For Willis has shown that an effervescence occurs when fresh blood is mixed with various liquids." The heart acts in the same way, and expels the blood not so much by contraction as by a swelling up of the walls of the ventricles, just as a bullet is shot from a gun by the expansion of the powder, and the forces employed are not dissimilar, for Borelli calculates that each heart-beat overcomes a resistance equal to 180,000 lbs.! He then applies the theory to the explanation of fevers. Such affections are not due, as the "chemists" assert, to an acid, alkaline, saline, or sulphurous state of the humours, for we may drink liquids of those kinds to any amount without causing fever; besides, "I saw at Pisa oil of sulphur injected into a dog's veins, and he was none the worse for it." Nor is abnormal heat the primary symptom, as the Galenists say, for it is itself a result of the increased cardiac activity and more rapid circulation of the blood. This Borelli attributes to some change in the nervous fluid, to explain which he adopts Wharton's theory that the glands are the excretory organs of the nervous system, and so finally concludes that the proximate cause of fever is a blocking up of the minute pores of these glands by some viscid substance. Fevers are to some extent self-limited diseases, for the increased blood flow tends to wash away the viscid matter and thus free the glands; and the alternate accumulation and removal explains the intermittences so common in febrile disorders. The rules of treatment are readily deducible from the theory. The physician should for the most part wait upon Nature, but he may do some good by giving fluid diet to dilute the humours, and by administering drugs such as nitre, which appear to dissolve glutinous substances, or bark to restore the "tone" of the solids. Bleeding, though so often employed, is contra-indicated, for the "materies morbi" is not in the blood but in the nervous system, and Borelli concludes, "If this theory of fever is not false and utterly out of the way, I doubt not that ingenious physicians will soon discover more certain remedies."

One of the most famous followers of Borelli was

Lorenzo Bellini, for thirty years (1663-93) professor at Pisa, during which period he published numerous works expounding and defending the doctrines of the mechanical school of medicine. Perhaps the most interesting and original part of his teaching is his theory of local stimulus, or, as we now call it, counter-irritation. The iatromechanics held that the great causes of disease are alterations in the elasticity or "tone" of the solids, or in the density of the fluids, which hinder the free movement of the latter, and give rise to local congestions or stagnation. One way of dealing with such a state is by bleeding, especially from an artery, which, as Bellini is at great pains to prove, tends to increase the velocity of the circulation. But he considers that the same end may often be attained by local stimulus, by cautery, blisters, rubefacient lotions, flagellation, &c. These agents produce a local contraction of the fibres, whereby the fluids are expressed from one part to another, while at the same time the local blood flow is accelerated, and tends to wash out any morbid matter.

How completely the minds of the mechanical physicians were dominated by Harvey's discovery may be seen in the works of the chief British representative of the school, Archibald Pitcairne, the friend of Bellini, and tutor of Boerhaave. The following are the fundamental definitions with which he commences his "Elementa Medicinæ Physico-Mechanica." Life is the circulation of the blood. Health is its free and painless circulation. Disease is an abnormal motion of the blood, either general or local. Like the English school generally, he is far more exclusively mechanical than are the Italians, and will hear nothing of ferments or acids even in digestion. This, he declares, is a purely mechanical process due to heat and pressure, the wonderful effects of which may be seen in Papin's recently invented "digester." That the stomach is fully able to comminute the food may be proved by the following calculation. Borelli estimates the power of the flexors of the thumb at 3,720 lb., their average weight being 122 grains. Now, the average weight of the stomach is 8 oz., therefore it can develop a force of 117,088 lb., and this may be further assisted by the diaphragm and abdominal muscles, the power of which, estimated in the same way, equals 461,219 lb.! Well may Pitcairne add that this force is not inferior to that of any millstone. Indigestion is due either to paralysis of the stomach, which is very rare, or to the presence of a viscid substance on its surface and between its fibres, preventing their proper contraction. The treatment indicated is to give purgatives or drugs which will remove this viscosity, and he recommends, among other things, the Elixir Proprietatis of Sylvius.

The calculations of the British iatro-mathematicians, however, were not always so wild as those of Borelli and Pitcairne. Thus Keill estimated the force of the heart beat at from five to nine ounces, while he also repeated and corrected some of the observations of Sanctorius. Another member of the school, Dr. Edward Barry, thought that the probable length of life might be calculated mathematically, on the supposition that the heart is a clock-like machine set to beat so many times. Suppose, for example, a person be endowed with an organ which will last seventy years, at an average rate of sixty beats per minute then, if by excesses and excitements he increases the rate to seventy-five beats, he will live, excluding accidents, exactly 56 years.

But, with all their love of calcucation, the disciples of Borelli soon saw that it was absurd to apply the strict rules of mathematics to the varied and complicated phenomena of disease, and so began to separate their theory from their practice, following in the latter the principles of a rational empiricism; and so marked is this distinction in the works of the two greatest members of the school, Baglivi and Boerhaave, that they may fairly be classed with our own Sydenham among the clinical or Hippocratic physicians of the age.

